# Oriented Graphenes from Plasma-Reformed Coconut Oil for Supercapacitor Electrodes

**DOI:** 10.3390/nano9121679

**Published:** 2019-11-25

**Authors:** Shailesh Kumar, Phil Martin, Avi Bendavid, John Bell, Kostya (Ken) Ostrikov

**Affiliations:** 1 School of Chemistry, Physics and Mechanical Engineering, Queensland University of Technology, Brisbane, Queensland 4000, Australia; shaileshiitb7@gmail.com (S.K.); j.bell@qut.edu.au (J.B.); 2 QUT-CSIRO Joint Sustainable Processes and Devices Laboratories, Lindfield, NSW 2070, Australia; 3 CSIRO Manufacturing, Lindfield, NSW 2070, Australia; Phil.Martin@csiro.au (P.M.); Avi.Bendavid@csiro.au (A.B.)

**Keywords:** plasma nanoscience, oriented graphenes, plasma production of nanomaterials

## Abstract

The utilization of vertical graphene nanosheet (VGN) electrodes for energy storage in supercapacitors has long been desired yet remains challenging, mostly because of insufficient control of nanosheet stacking, density, surface functionality, and reactivity. Here, we report a single-step, scalable, and environment-friendly plasma-assisted process for the fabrication of densely packed yet accessible surfaces of forested VGNs (F-VGNs) using coconut oil as precursor. The morphology of F-VGNs could be controlled from a continuous thick structure to a hierarchical, cauliflower-like structure that was accessible by the electrolyte ions. The surface of individual F-VGNs was slightly oxygenated, while their interior remained oxygen-free. The fabricated thick (>10 μm) F-VGN electrodes presented specific capacitance up to 312 F/g at a voltage scan rate of 10 mV/s and 148 F/g at 500 mV/s with >99% retention after 1000 cycles. This versatile approach suggests realistic opportunities for further improvements, potentially leading to the integration of F-VGN electrodes in next-generation energy storage devices.

## 1. Introduction

The global interest in nanomaterial-enabled energy storage devices is growing to fulfill the requirement to power future renewable energy storage systems [1,2,3]. Supercapacitors, also known as electrochemical capacitors, feature higher power density, faster charge and discharge, and longer life cycle over conventional batteries [4,5,6]. However, real-world applications of supercapacitors need to satisfy several criteria, such as high specific capacitance, stable and reliable charge, and discharge with near-complete capacitance retention [7,8,9].

Vertically oriented graphene nanosheets, also termed vertical graphene nanosheets (VGNs) or vertical graphenes (VGs) for simplicity, hold great promise for high-performance supercapacitor electrodes. VGNs are commonly synthesized using low-temperature plasmas and offer unusual electrical and mechanical properties. As such, VGNs represent versatile building blocks with high density of active edges and large surface areas, which are particularly favorable for the development of three-dimensional (3D) networks of advanced electrode materials for charge storage devices [10,11,12,13,14,15,16,17,18,19]. However, despite almost two decades of intense research efforts since the pioneering reports on VGNs in early 2000s [20,21], their application in high-performance supercapacitors remains challenging. 

One of the key reasons is due to the limited control over the VGN morphology, stacking, density, and surface functionality during the fabrication process. In particular, vertical stacking of multiple layers of VGNs is favorable when trying to achieve high mass loading of VGNs per surface area of electrode materials. However, such multilevel, dense stacking of VGNs limits ideal access to all the surfaces of all the stacked VGNs by the electrolyte ions as well as the ability of the ions to move in and out of the VGN stack during the charge–discharge cycles, especially at high voltage scan rates [22,23].

In this work, we report a single-step, scalable, and environment-friendly plasma-assisted process for the fabrication of densely packed yet highly accessible surfaces of forested VGNs (F-VGNs) using coconut oil as precursor. The fabricated vertically oriented graphene structures reveal functionality for energy storage and, with further optimization, are potentially applicable for supercapacitor electrodes.

## 2. Materials and Methods 

### 2.1. Synthesis of Forested Vertical Graphene Nanosheet Structures 

The fabrication of VGNs was conducted in radio-frequency (RF, 13.56 MHz) inductively coupled plasma-assisted chemical vapor deposition (CVD) system operated at RF power input up to 1 kW. Cheap and easily available coconut oil was used as carbon precursor for the process. Only a few drops of oil (up to ~2 mL) were drop-cast on a large sample (>3.5 × 3.5 cm^2^) of porous Ni foam with a porosity of >93%, which was used as substrate. After loading the substrate into the CVD chamber, Ar, H_2_, and N_2_ gases were fed into the chamber via mass flow controllers for the deposition of thick cauliflower-like F-VGNs. The growth of F-VGNs was carried out at RF power of up to 1000 W for 8 min. During the process, the chamber pressure was kept constant in the ~40–80 mTorr range. The substrate temperature reached up to ~350–450 °C due to plasma heating. No external heating source was used prior to or during deposition. During the growth phase with methane gas as precursor, VGNs with continuous morphology were deposited; this process did not require nitrogen gas addition or oil precursor. 

### 2.2. Microscopy and Microanalysis

A field-emission scanning electron microscopy (FE-SEM; Zeiss, Auriga, Oberkochen, Germany with an in-lens secondary electron detector was operated at an electron beam energy of 5 keV to study the morphology of deposited F-VGNs. A Renishaw in via spectrometer (514 nm laser source) and transmission electron microscopy (TEM)/high-resolution TEM (HRTEM) (JEOL 2100 200kV accelerating voltage; JEOL Ltd., Tokyo, Japan) were used for structural characterization. The surface composition of the samples was assessed by X-ray photoelectron spectroscopy (XPS; SPECS Surface Nano Analysis GmbH, Berlin, Germany) using a SPECS SAGE 150 instrument operated with Mg K excitation at 1253.6 eV. 

A BioLogic VSP 300 potentiostat/galvanostat (BioLogic Science Instruments, Seyssinet-Pariset, France) was used for the electrochemical measurements using 0.1 M Na_2_SO_4_ aqueous solution at room temperature. A three-electrode cell configuration with F-VGN electrode as working electrode, Pt wire mesh (2.0 × 2.0 cm^2^) as counter electrode, and Ag/AgCl as reference electrode was used for the measurements. Cyclic voltammetry (CV) tests were swept in the potential window of 0 to 0.8 V. Galvanostatic charge–discharge curves were obtained at a constant current density of 1.5 A/g. Electrochemical impedance spectroscopy (EIS) measurements were carried out in a frequency range from 10 Hz to 100 kHz. The specific capacitance C was calculated using CV curves by following a standard procedure [16,17], which involved integrating the discharge current (I) with potential (V) and taking into account the scan rate (V/s), the mass of the electrode material (m), and the operating potential window 0.8 V.

## 3. Results

### 3.1. Fabrication, Characterization, and Features of VGNs 

**Figure 1:**
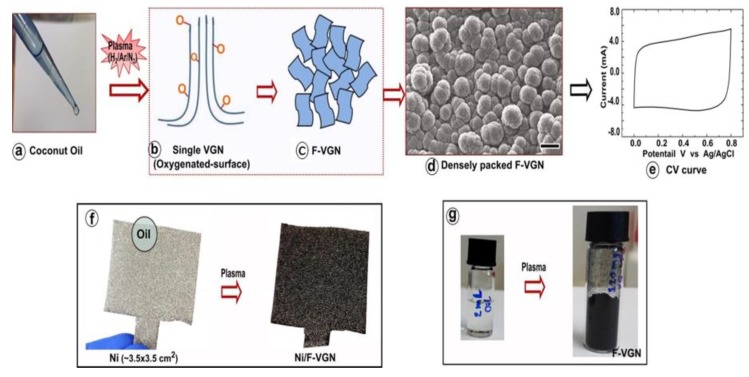
Growth process of forested vertical graphene nanosheets (F-VGNs) in a plasma chemical vapor deposition (CVD) system. (**a**) Small drops of coconut oil were used as precursor. (**b,c**) Schematic diagram of formation of individual VGNs with oxygenated surfaces and densely packed, cauliflower-like structure. (**d**) A scanning electron microscopy (SEM) image of as-deposited F-VGNs. (**e**) A representative cyclic voltammetry (CV) curve of F-VGN electrode at a voltage scan rate of 500 mV/s in a dilute (0.1 M) aqueous solution of Na_2_SO_4_. (**f**) Ni foam before and after deposition of F-VGNs. (**g**) Only 2 mL of coconut oil was used to produce ~120 mg of F-VGNs.

Densely packed forested VGNs were synthesized using a reactive plasma-assisted CVD process, with coconut oil (fatty acids) used as a precursor for the carbon source (Figure 1a–c). A gas mixture of Ar + H_2_ + N_2_ was introduced into the reaction chamber for 8 min of the growth process. During the growth of the VGN, the surface of the VGN was also slightly oxygenated. Moreover, the VGNs self-organized into cauliflower-like hierarchical structures during the growth process, as can be seen in Figure 1.

The electrochemical performance of deposited F-VGN film was tested at different scan rates. An almost rectangular shape of CV curve (Figure 1d) was observed, even at a high voltage scan rate of 500 mV/s in a dilute solution of Na_2_SO_4_ (0.1 M). This result suggests the electrical double-layer capacitance (EDLC) mechanism of the F-VGN electrode as well as the possibility of short diffusion paths for the electrolyte ions to reach the VGN surfaces, even deep inside the hierarchical VGN stack. We emphasize that the thickness of F-VGN film as an active electrode was about 10 μm. 

The growth of F-VGNs using oil (in this case coconut oil) is sustainable and scalable. A small amount of oil (~200 μL) was used to deposit a uniform film of F-VGNs with thickness >10 μm on the 3.5 × 3.5 mm^2^ area of a commercial Ni foam (thickness of the foam was ~1 mm). The plasma-based process produced about 120 mg of F-VGN powder using only 2 mL of coconut oil (Figure 1e,f). The process may be further improved and optimized to produce even higher amounts of F-VGNs per mL of oil precursor. 

**Figure 2:**
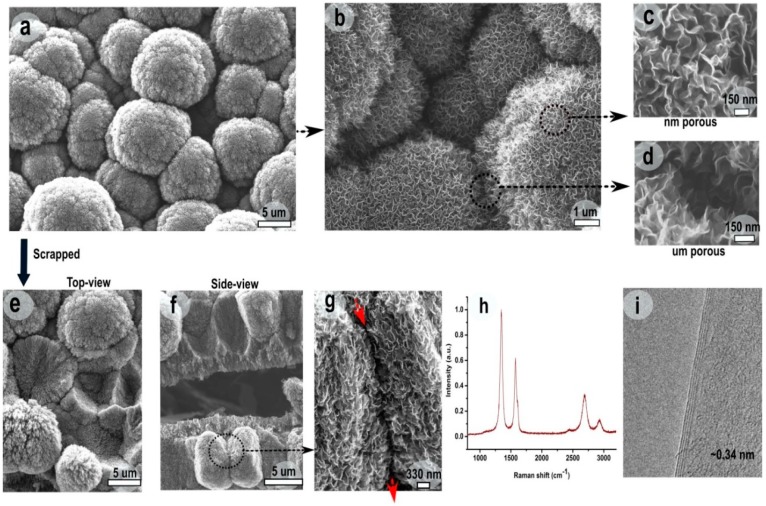
Morphological and structural characterization of F-VGNs: (**a–g**) SEM images, (**h**) Raman spectrum, and (**i**) transmission electron microscopy (TEM) image.

A detailed morphological analysis of densely packed, cauliflower-like F-VGNs was performed using FE-SEM analysis (Figure 2a–g). The size of individual cauliflower-like F-VGNs in the film varied from 1–10 μm in diameter. While the cauliflowers were connected in a mesh-like network, they were also separated sufficiently from each other (marked with arrows in Figure 2a,b). The width of the gap or void between two neighboring cauliflowers was of the order of 6 μm. Further high-resolution SEM images (Figure 2b–d) showed that the gap between two cauliflower-like beads made of VGN “bunches” was typically as small as ~50–150 nm. However, the gaps between two individual VGNs within a single cauliflower-like bead were much smaller, typically well under 50 nm, yet sufficiently large to facilitate effective ion transport. 

The fabricated hierarchical structure was further investigated by mechanically scraping the film using tweezers. Top and side views of the scrapped film (Figure 2d,f) suggested that cauliflower-like beads were stacked, forming a dense, three-dimensional stack. Importantly, neighboring stacks of the cauliflower-like beads formed channels varying in width from ~50 to 490 nm, as indicated by red arrows in Figure 2g. The figure also reveals that individual cauliflower-like beads were formed by bunches of stacked graphene flakes. The resulting hierarchical structure thus had pores that connected its interior to the outer surface. 

These channels were made of larger channels between the densely packed, cauliflower-like beads and a network of smaller channels between F-VGNs within the same cauliflower. This hierarchical internetwork structure created effective channels connecting the top surface to the interior of the film, thereby providing accessible paths for electrolyte ions to effectively enter and exit the supercapacitor electrode during the operation. Such morphology, with ion transport channels interconnected at both nanometer and microscopic scales, is highly desirable for designing high-performance electrodes for energy storage devices. The electrodes therefore featured very large active surface areas made up of all the surfaces presented by the VGNs in the hierarchical stack. 

Raman spectroscopy and TEM were used to further investigate the structure of as-deposited F-VGNs. Figure 2h shows a Raman spectrum of the electrode material at room temperature. The spectrum shows three intense Raman peaks: a sharp graphitic G peak (~1585 cm^−1^), a second-order 2D peak (~2705 cm^−1^), and a disorder-related D peak (~1351 cm^−1^) [24,25,26]. The relative intensity of the 2D peak with respect to the G peak along with the shape of the 2D peak is also an indication of the number of graphene layers; the 2D/G intensity ratio varied from 0.5 to 0.7, whereas the full width at half maximum (FWHM) of the 2D peak varied between 58 and 64 cm^−1^. The low FWHM indicates that the individual VGNs were composed of less than six graphene layers. This conclusion was also confirmed by HRTEM (Figure 2i), which revealed the presence of only four graphene layers in the selected graphene flake. The distance between the two graphene layers was found to be ~0.34 nm. Apart from the presence of defects, the D peak involved contribution from the open edges in the stacked graphene layers.

**Figure 3:**
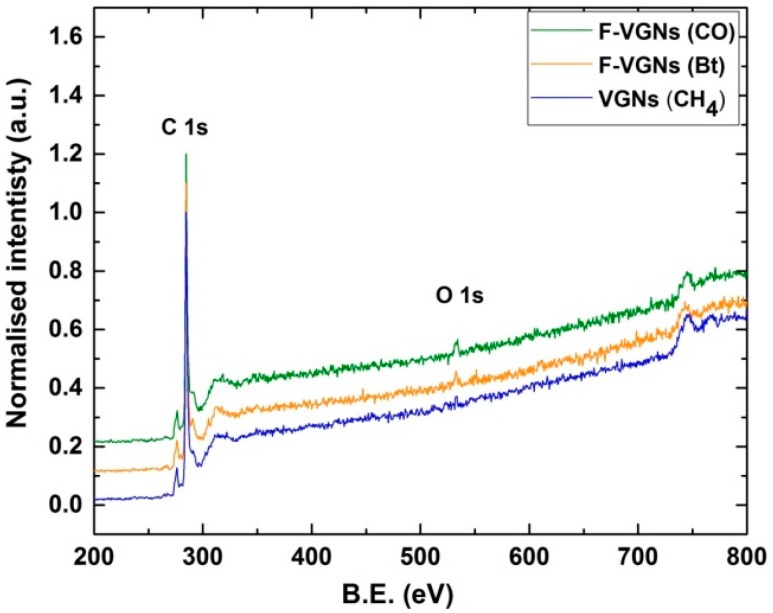
Surface chemical analysis of VGNs. X-ray photoelectron spectroscopy (XPS) curves of F-VGNs from coconut oil (green), butyric acid (orange), and methane (blue). The notations in the box on the top right denote precursors used to fabricate the nanostructures, namely, CO—coconut oil, Bt—butyric acid, and CH_4_—methane.

XPS measurements (Figure 3) suggested that the oxygen content in the F-VGNs during the growth could be varied from ~0.5 to ~5 at % (XPS data is given in atomic percent, at %) by simply using different carbon precursors. The coconut oil-produced F-VGNs were not only self-organized in densely packed, cauliflower-like structures but also slightly functionalized with oxygen (up to ~5 at %). It was also observed that the oxygen content was almost the same, even for the VGNs formed during an early growth stage of only 3 min. However, butyric acid-produced cauliflower-like F-VGN films showed only 1.2 at % oxygen content. Furthermore, the oxygen content was very low (only ~0.2 at %) in the methane-produced F-VGN film. It should be noted that no external oxygen gas source was used during the growth process, indicating that the oil was the likely source of oxygen.

Figure 3 shows the presence of carbon and oxygen atoms on the surface of F-VGNs grown using coconut oil, butyric acid, and methane. The main observation is that the presence of O atoms on the surface correlated with the oxygen content in the carbon precursors. The very small oxygen content on the surface of the sample produced from (oxygen-free) purified methane was most likely due to the residual oxygen in the chamber.

### 3.2. Electrochemical Properties of the Electrodes

**Figure 4:**
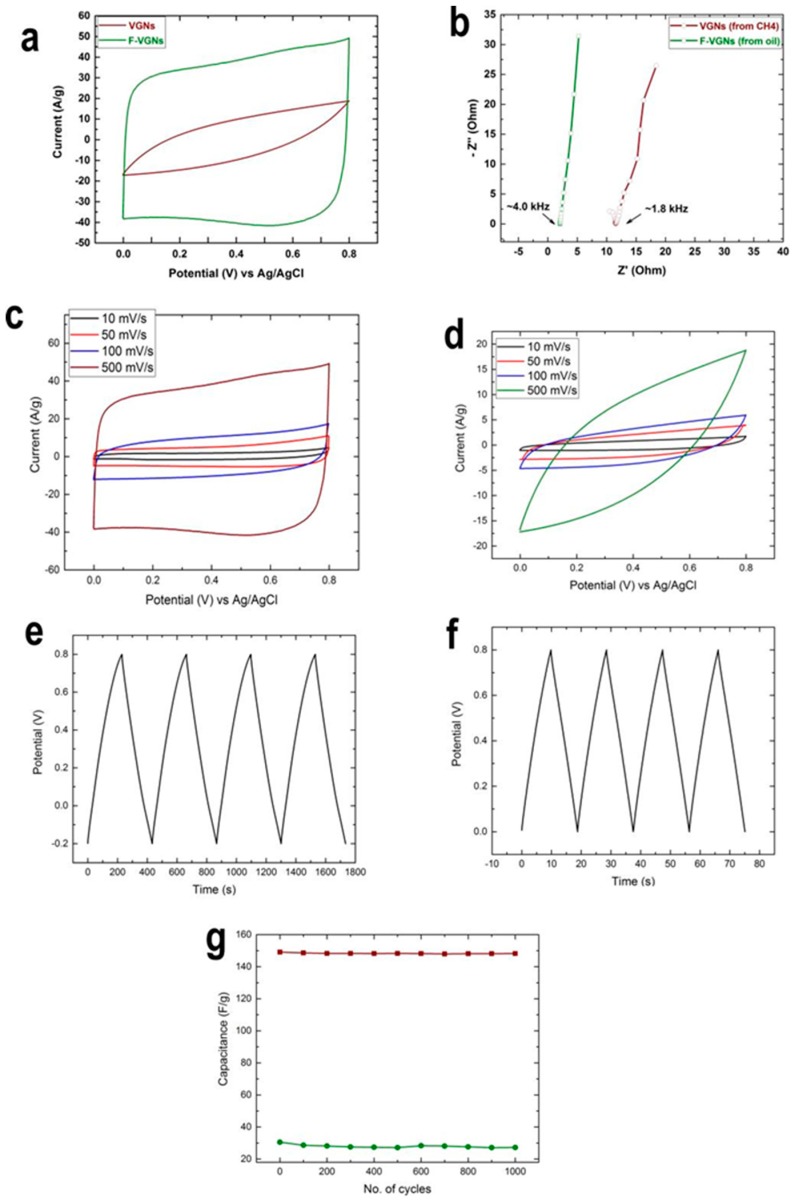
Electrochemical performance of F-VGN and VGN electrodes derived from coconut oil and methane, respectively, in a dilute (0.1 M) aqueous solution of Na_2_SO_4_. (**a**) CV curves of the F-VGN and VGN samples at a scan rate of 500 mV/s. (**b**) Nyquist plots for the F-VGN and VGN samples. (**c,d**) CV curves for F-VGN and VGN samples, respectively, at different scan rates. (**e,f**) Charge and discharge curves at a constant current of 1.5 A/g for F-VGN and VGN samples, respectively. (**g**) Specific capacitance and cycle stability of the F-VGN and VGN samples at 500 mV/s.

The electrochemical performance of the F-VGN structures was evaluated in a conventional three-electrode cell configuration. Figure 4a shows the CV curves of F-VGNs (of 10 μm thickness) and VGNs (of 5 μm thickness) grown from the oil and methane precursors, respectively, at a high voltage scan rate of 500 mV/s in aqueous solution of 0.1 M Na_2_SO_4_. The CV curve corresponding to the F-VGN electrode showed a nearly rectangular shape, suggesting electric double-layer formation as well as effective ion transport through the interconnected channel network in the electrode material. However, the corresponding shape for the VGN electrode was oval-like (rather than closer to a rectangular shape), suggesting that further improvement in the efficacy of the electrostatic double-layer energy storage mechanism might be needed. One can clearly notice that the current density for the F-VGN sample was almost five times higher than that for the VGN sample. In other words, easily accessible, larger graphene surface and effective ion transport channels in the F-VGNs sample indeed improved the energy storage capacity and charge–discharge kinetics of the electrode material. 

Figure 4c,d shows the CV curves collected at a different scan rates from the F-VGN and VGN samples, respectively. This data reveals that the effective EDL formation took place for all (from low to high) voltage scan rates for the F-VGN samples. On the contrary, the EDL performance was much reduced for the VGN samples at the scan rates of 50 mV/s or above. To the best of our knowledge, this range of consistently sustained high current upon EDL formation at a high voltage scan for a thick (>10 μm) densely packed, vertically oriented graphene-based electrode has never been reported. 

Figure 4 shows the charge and discharge curve of the two electrodes at a current density of 1.5 A/g. A linear dependence of discharge potential with respect to time implies the absence of any major Faradaic processes. Figure 4b shows the electrochemical impedance spectra (EIS) for the electrodes. The frequency-dependent impedance is given as real (Z’) and imaginary (Z’’) components in the Nyquist plot. A vertical curve featured at low frequencies indicates the almost-ideal capacitive behavior of both electrodes. It can also be noticed that a semicircle intersected with the real (Z’) axis in the high-frequency range, which indicates charge transfer at the electrode–electrolyte interface. The intersection was lower (2.1 Ohm) in F-VGNs than VGNs (12.1 Ohm), which indicates lower charge transfer resistance in F-VGNs than VGNs. These values of electrical resistance in F-VGNs are comparable to other graphene-based electrodes prepared by conventional chemical or thermal methods. These conclusions are consistent with the results of previous studies on vertical graphene and similar structures [27,28].

The specific capacitance (SC) of the electrode, as shown in Figure 4g, was calculated using the CV curves by integrating discharge current over potential (V). While the specific capacitance for VGNs was 28 F/g at a given scan rate of 500 mV/s, it drastically increased to 148 F/g for F-VGNs. This value is comparable to the capacitances typically obtained with other carbon-based nanostructured materials. Furthermore, the SC reached 189 F/g at the scan rate of 100 mV/s and 312 F/g at the scan rate of 10 mV/s. This value is among the best graphene-based electrodes produced by CVD and is superior to the specific capacitance for thick and densely packed VGN films (>5 μm). The cycling tests for capacitance in Figure 4g show that both samples retained >99% of their initial capacitance after 1000 cycles at a scan rate of 500 mV/s. These results therefore demonstrate a reliable electrochemical performance of the F-VGN electrodes. Further studies are envisaged in the future to quantify the rate capabilities as well as performance stability using longer scanning periods. The relationship of the energy storage capacity and kinetics to the structure and density of the microporous structure represents another interesting opportunity for research.

## 4. Conclusions

In summary, we have demonstrated a simple, single-step, and potentially scalable plasma-assisted process for deposition of densely packed yet well-accessible surfaces of forested VGNs. The morphology of F-VGNs could be effectively controlled from a semicontinuous film-like structure to a highly ion-accessible hierarchical cauliflower-like structure. Moreover, the surface of individual F-VGNs was slightly oxygenated (~4.6% by *w*/*w*), whereas the inner graphene layers in the individual nanosheets remained essentially oxygen-free. The fabricated thick (>10 μm) F-VGN cauliflower-like electrodes demonstrated specific capacitance up to 312 F/g at a voltage scan rate of 10 mV/s and 148 F/g at 500 mV/s with >99% retention after 1000 cycles. This effective method for fabricating F-VGN electrodes with a promising capacitance >100 F/g at high scan rates paves the way, with further optimization, for the direct integration of VNGs into advanced supercapacitor devices.
